# An IncP-2 plasmid sublineage associated with dissemination of *bla*_IMP-45_ among carbapenem-resistant *Pseudomonas aeruginosa*

**DOI:** 10.1080/22221751.2021.1894903

**Published:** 2021-03-13

**Authors:** Xuefei Zhang, Leilei Wang, Dan Li, Pei Li, Lili Yuan, Fan Yang, Qinglan Guo, Minggui Wang

**Affiliations:** a Institute of Antibiotics, Huashan Hospital, Fudan University, Shanghai, People’s Republic of China; b Infection Control Unit, Huashan Hospital, Fudan University, Shanghai, People’s Republic of China

**Keywords:** Carbapenem-resistant *Pseudomonas aeruginosa*, outbreak, *bla*_IMP-45_, IncP-2 plasmid, sublineage, dissemination

## Abstract

IMP-45, a variant of IMP-9, is one of the dominant metallo-β-lactamases (MBLs) in clinical carbapenem-resistant *Pseudomonas aeruginosa* (CRPA) isolates in China. The aim of this study was to investigate the distribution and mechanism of dissemination of *bla*_IMP-45_. MBL genes were detected by PCR in 173 non-duplicate CRPA isolates collected from Hospital HS in Shanghai and 605 *P. aeruginosa* isolates from a multicenter surveillance of *bla*_IMP-45_ in China. In total, 17 IMP-45-producers (14 from Hospital HS and 3 from other hospitals) were identified. Molecular typing identified an outbreak of 11 IMP-45-producing ST508 CRPA in the ICU of Hospital HS. Conjugation assays and whole genome sequencing were conducted among IMP-45-producers. Genomic comparison revealed that 16 *bla*_IMP-45_-carrying plasmids (9 from this study and 7 from GenBank) shared a similar backbone with IncP-2 *bla*_IMP-9_-carrying plasmid pOZ176 but lacked *repA*-*oriV*-*parAB* region. *repA2* gene was presented in pOZ176, *bla*_IMP-45_-carrying plasmids (17 from this study and 7 from GenBank) and 15 megaplasmids from GenBank. Phylogenetic analysis of *repA2* showed that most *bla*_IMP-45_-carrying plasmids were clustered into a sublineage separate from the one containing pOZ176. This IncP-2 plasmid sublineage contributed to the dissemination of *bla*_IMP-45_ among genetically diverse *P. aeruginosa* and recruited multiple resistance genes during its evolution.

## Introduction

*Pseudomonas aeruginosa* is prone to be resistant to β-lactams, aminoglycosides and quinolones. Production of metallo-β-lactamases (MBLs) is one of the primary carbapenem resistance mechanisms in this species, among which IMP and VIM are the most prevalent [1]. IMP-1, IMP-4, IMP-6, IMP-8, IMP-9, IMP-10 and IMP-45 have been reported in China [2]. IMP-9 was initially identified in *P. aeruginosa* isolates from Guangzhou, China [3]. Afterwards, outbreaks of IMP-9-producing *P. aeruginosa* were observed in this area in 2000 and from 2005 to 2007 [3,4]. IMP-45, a single amino acid substitution variant (G214S) of IMP-9, was first reported in a canine-origin *P. aeruginosa* from Beijing, China in 2014, showing higher level resistance to meropenem than to imipenem [5]. Afterwards, more clinical isolates of IMP-45-producing *Pseudomonas* were discovered [6–8], including one isolated from a French patient who had been repatriated from an ICU in Shanghai, China [8], implying the risk of worldwide spread of *bla*_IMP-45_ by such dissemination.

IMP-9-encoding plasmid pOZ176 (500 kb), the only full sequenced IncP-2 plasmid before 2013, contained *bla*_IMP-9_, *bla*_OXA-10_ and *aacA4* genes conferring β-lactam and aminoglycoside resistance. Two replication genes, *repA* and *repA2* were identified in pOZ176 [3,9]. IncP-2 plasmids, generally >300 kb and in single copy, exhibit tellurite resistance and are narrow-host-range for *Pseudomonas spp* [10]. Previous studies have characterized two *bla*_IMP-45_-carrying megaplasmids from clinical isolates (*P. putida* and *P. aeruginosa*) in China [6,7], but information about the dissemination of *bla*_IMP-45_ gene remains largely limited and the role of plasmids in the dissemination of *bla*_IMP-45_ is poorly understood.

In this study, we report an outbreak of carbapenem-resistant *P. aeruginosa* (CRPA) co-carrying *bla*_IMP-45_, *qnrVC1* and *armA* in a tertiary hospital of Shanghai and the disappearance of outbreak clones after strengthened infection control measures. Subsequently, we carried out a multicenter surveillance of *bla*_IMP-45_ in *P. aeruginosa* clinical isolates in China and explored the role of IncP-2 plasmids in the dissemination of *bla*_IMP-45_ among *P. aeruginosa*.

## Material and methods

### Clinical isolates and antimicrobial susceptibility testing

One hundred and seventy-three non-duplicate CRPA isolates were collected from a tertiary hospital (Hospital HS) in Shanghai between January 2015 and April 2018. CRPA was defined as *P. aeruginosa isolate* resistant to either imipenem or meropenem. Additionally, a multicenter surveillance was performed with 605 non-duplicate *P. aeruginosa* isolates collected consecutively from 11 hospitals in 8 provinces/municipalities across China, including 3 hospitals in Shanghai, 2 in Beijing and 1 in each of the other 6 provinces (July, 2018 to February, 2019, Supplementary Table S1).

Minimal inhibitory concentrations (MICs) were determined for 13 antimicrobial agents by broth microdilution method and interpretation was according to recommendations of the CLSI [11].

### MBLs screening and identification

PCR amplification was performed to screen for MBL genes (*bla*_IMP_, *bla*_VIM_, *bla*_NDM_, *bla*_SPM_, *bla*_SIM_, *bla*_GIM_, *bla*_DIM_, *bla*_AIM_ and *bla*_FIM_) [12,13]. *bla*_IMP_-positive isolates were further amplified with primers specific for various subtypes (Supplementary Table S2).

### Molecular typing of *P. aeruginosa* isolates

Pulsed-field gel electrophoresis (PFGE) was performed using SpeI (TaKaRa Bio, Dalian, China) as the restriction enzyme and with a switch time of 2 s-40 s [14,15]. The PFGE patterns were analysed by BioNumerics (version 4.0; Applied Maths, Inc.) and a dendrogram was generated by the UPGMA method based on the Dice coefficient.

Multilocus sequence typing (MLST) was performed according to the instructions in the *P. aeruginosa* MLST website (http://pubmlst.org/paeruginosa/). STs were compared with those in the MLST *P. aeruginosa* database by goeBURST [16]. A neighbor-joining tree from concatenated seven housekeeping genes (*acsA*, *aroE*, *guaA*, *mutL*, *nuoD*, *ppsA*, and *trpE*) was generated using MEGA 7.0 [17].

### Conjugation assay

Transfer of plasmid carrying *bla*_IMP-45_ was performed by filter mating with *P. aeruginosa* PAO1^Rif^ (rifampin resistant) as recipient. Transconjugants were selected on LB plates supplemented with meropenem (2 mg/L) and rifampin (500 mg/L), and further confirmed by PCR amplification and sequencing of *bla*_IMP-45_ and *guaA*, one of the seven housekeeping genes for MLST of *P. aeruginosa*. The transconjugants harbouring *bla*_IMP-45_ were tested for antimicrobial susceptibility. Conjugation was also carried out using *Escherichia coli* J53 (azide resistant) as recipient.

### Whole genome sequencing (WGS) and sequence assembly

Sequencing of 9 IMP-45-producers, including 6 *P. aeruginosa* clinical isolates (HS15-106, HS17-127, HS18-41, GZ18-2, KM18-18 and RJ19-28) and 3 transconjugants (TcHS15-101, TcHS15-158 and TcHS15-172) were performed on Hiseq X-ten or Novaseq platforms (Illumina Inc., San Diego, CA, USA). De novo assembly was performed using Velvet version 1.2.03 [18] or SOAPdenovo2 [19]. To obtain the complete genomic sequence, clinical isolate of *P. aeruginosa* HS17-127 was further subjected to Pacbio sequencing and assembled with HGAP [20].

For the 8 transconjugants of outbreak isolates without WGS data, the *repA2* gene and genetic context surrounding *bla*_IMP-45_ were amplified by PCR and sequenced with a series of primers designed according to the genome sequence of the transconjugant of *P. aeruginosa* outbreak isolate HS15-101 (Table S2).

### Bioinformatics analysis

The contigs were annotated using RAST (http://rast.nmpdr.org/), screened for insertion sequences with ISfinder [21], and analysed for STs, antibiotic resistance genes and plasmid typing at the Centre for Genomic Epidemiology web site (http://www.genomicepidemiology.org/). BLASTN searches were conducted to find the complete sequenced *bla*_IMP-45_-harbouring plasmids in GenBank database using *bla*_IMP-45_ as the query sequence. BLAST Ring Image Generator (BRIG) [22] were used in the comparative analysis of the plasmids.

### Nucleotide sequence accession numbers

The sequences of transconjugants TcHS15-101, TcHS15-158 and TcHS15-172, and *P. aeruginosa* clinical isolates HS15-106, HS17-127, HS18-41, GZ18-2, KM18-18 and RJ19-28 were submitted to GenBank with Bioproject ID PRJNA631492 (Supplementary Table S3). The accession numbers for the chromosome of *P. aeruginosa* clinical isolate HS17-127 and plasmid pHS17-127 are CP061376 and CP061377, respectively.

## Results

### Outbreak of bla_IMP-45_-bearing *P. aeruginosa* in Hospital HS

Fourteen out of 173 CRPA from Hospital HS were positive for *bla*_IMP-45_: 12 CRPA isolated from 2015, 1 from 2017 and 1 from 2018. All of them exhibited resistance to antipseudomonal β-lactams excluding aztreonam, β-lactamase inhibitor combinations and aminoglycosides. The MICs of aztreonam ranged from 4 to 16 mg/L in 7 strains whereas the remaining strains were highly resistant to aztreonam (from 64 and >128 mg/L). All the *bla*_IMP-45_-carriers were resistant to quinolones except isolate HS17-127 (Table 1).

**Table 1.** Characteristics of 17 *Pseudomonas aeruginosa* clinical isolates carrying *bla*_IMP-45_.

Clinical isolate	Source	Transconjugant	Sequence type	MICs (mg/L)^b^													Resistance gene
				PIP	TZP	CAZ	CZA	FEP	ATM	IPM	MEM	CIP	LEV	AMK	CT	RIF	
HS15-101	Urine	TcHS15-101	ST508	128	128	>128	>128	>128	8	32	>128	8	16	>128	1	256	*bla*_IMP-45_, *bla*_OXA-1_, *armA*, *qnrVC1*
HS15-106	Sputum	Failed	ST3014	64	64	>128	>128	128	8	32	>128	4	8	>128	0.5	256	*bla*_IMP-45_, *bla*_OXA-1_, *armA*, *qnrVC1*
HS15-109	sputum	TcHS15-109	ST508	128	128	>128	>128	>128	8	32	>128	8	16	>128	0.5	384	*bla*_IMP-45_, *bla*_OXA-1_, *armA*, *qnrVC1*
HS15-110	sputum	TcHS15-110	ST508	128	128	>128	>128	>128	64	32	>128	16	64	>128	0.5	512	*bla*_IMP-45_, *bla*_OXA-1_, *armA*, *qnrVC1*
HS15-111	sputum	TcHS15-111	ST508	128	128	>128	>128	>128	32	64	>128	16	64	>128	1	512	*bla*_IMP-45_, *bla*_OXA-1_, *armA*, *qnrVC1*
HS15-141	sputum	TcHS15-141	ST508	128	128	>128	>128	128	8	32	>128	8	16	>128	0.5	384	*bla*_IMP-45_, *bla*_OXA-1_, *armA*, *qnrVC1*
HS15-146	sputum	TcHS15-146	ST508	>128	>128	>128	>128	>128	>128	64	>128	16	64	>128	0.5	512	*bla*_IMP-45_, *bla*_OXA-1_, *armA*, *qnrVC1*
HS15-158	sputum	TcHS15-158	ST508	>128	>128	>128	>128	>128	>128	64	>128	16	64	>128	0.5	512	*bla*_IMP-45_, *bla*_OXA-1_, *armA*, *qnrVC1*
HS15-172	sputum	TcHS15-172	ST508	>128	>128	>128	>128	>128	>128	64	>128	16	64	>128	0.5	512	*bla*_IMP-45_, *bla*_OXA-1_, *armA*, *qnrVC1*
HS15-176	sputum	TcHS15-176	ST508	128	128	>128	>128	>128	8	32	>128	8	16	>128	0.5	256	*bla*_IMP-45_, *bla*_OXA-1_, *armA*, *qnrVC1*
HS15-209	sputum	TcHS15-209	ST508	128	128	>128	>128	>128	16	64	>128	8	16	>128	0.5	512	*bla*_IMP-45_, *bla*_OXA-1_, *armA*, *qnrVC1*
HS15-215	urine	TcHS15-215	ST508	>128	>128	>128	>128	>128	64	32	>128	16	64	>128	0.5	512	*bla*_IMP-45_, *bla*_OXA-1_, *armA*, *qnrVC1*
HS17-127	sputum	TcHS17-127	ST369	128	128	>128	>128	>128	64	128	128	1	2	>128	0.5	32	*bla*_IMP-45_, *bla*_OXA-1_, *bla*_PER-1_, *bla*_AFM-1_, *armA*, *qnrVC6*
HS18-41	sputum	TcHS18-41	ST357	128	128	>128	>128	>128	4	32	>128	4	8	16	1	384	*bla*_IMP-45_, *bla*_OXA-1_, *armA*, *qnrVC1*
KM18-18^a^	sputum	TcKM18-18	ST274	128	128	>128	>128	128	4	64	>128	16	32	>128	0.5	512	*bla*_IMP-45_, *bla*_OXA-1_, *armA*, *qnrVC1*
GZ18-2^a^	sputum	Failed	ST1420	64	64	>128	>128	128	16	2	8	64	128	>128	1	384	*bla*_IMP-45_, *bla*_OXA-1_, *armA*, *qnrVC1*
RJ19-28^a^	wound fluid	TcRJ19-28	ST708	>128	128	>128	>128	>128	>128	>128	>128	16	16	>128	1	16	*bla*_IMP-45_, *bla*_OXA-1_, *bla*_AFM-1_, *qnrVC6*

^a^ *bla*_IMP-45_-carrying *P. aeruginosa* isolates from multicenter surveillance.

^b^ PIP: piperacillin; TZP: piperacillin/tazobactam; CAZ: ceftazidime; CZA: ceftazidime/avibactam; FEP: cefepime; ATM: aztreonam; IPM: imipenem; MEM: meropenem; CIP: ciprofloxacin; LEV: levofloxacin; AMK: amikacin; CT: colistin; RIF: rifampin.

**Figure 1.**
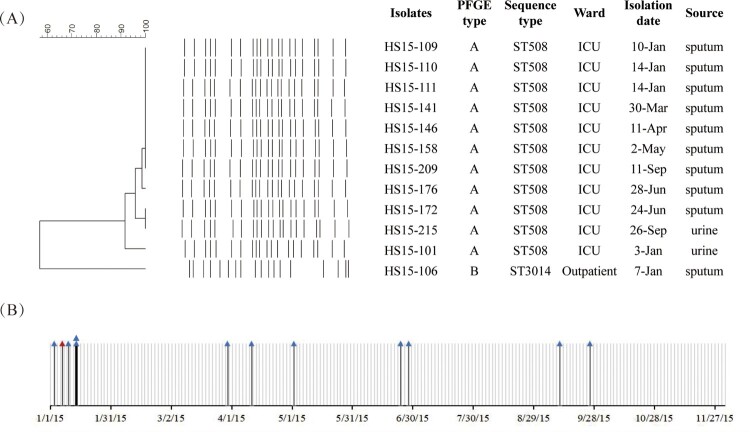
Twelve carbapenem-resistant *Pseudomonas aeruginosa* (CRPA) isolates carrying *bla*_IMP-45_ at Hospital HS in 2015. (A) PFGE of 12 *bla*_IMP-45_-bearing CRPA isolates. ST508 isolates belonged to pattern A and HS15-106 typed as pattern B. (B) Timeline of the first positive cultures of the *bla*_IMP-45_-bearing CRPA isolates for the 12 patients. The blue triangle indicates the isolation date of the *bla*_IMP-45_-carrying *P. aeruginosa* from ICU patients and the red indicates that of the outpatient.

Eleven out of 12 *bla*_IMP-45_-bearing CRPA from 2015 were isolated from ICU patients and shared the same sequence type, ST508 (Figure 1(A)). They presented indistinguishable or closely related PFGE patterns, different from that of HS15-106 (ST3014) from an outpatient. All the 12 *bla*_IMP-45_-bearing CRPA from 2015 were discovered in the first nine months this year as indicated by the timeline of the isolation date of the first *bla*_IMP-45_-carrying *P. aeruginosa* from each patient (Figure 1(B)). Taken together, an outbreak of *bla*_IMP-45_-bearing ST508 CRPA occurred in the ICU of Hospital HS in 2015. The other two *bla*_IMP-45_-bearing CRPA, HS17-127 (ST369) and HS18-41 (ST357), were clonally distinct from the previous outbreak *bla*_IMP-45_-carriers in Hospital HS.

### Multicenter surveillance of bla_IMP-45_

To investigate the prevalence of *bla*_IMP-45_, a multicenter surveillance was carried out among 605 *P. aeruginosa* clinical isolates collected throughout China, including 226 CRPA. Genotypic characterization found 8 isolates positive for *bla*_IMP_ (4 isolates positive for *bla*_IMP-14-like_, 3 positive for *bla*_IMP-45_ and 1 for *bla*_IMP-3_) and 6 isolates carrying *bla*_VIM-2_. No additional MBL-producing isolates were found. These 3 *bla*_IMP-45_-carriers belonged to ST1420, ST274 and ST708, respectively (Table 1).

### Transferability of bla_IMP-45_

To examine the transferability of *bla*_IMP-45_, all the 17 *bla*_IMP-45_-carrying CRPA were performed conjugation with *P. aeruginosa* PAO1^Rif^ as recipients. Fifteen transconjugants harbouring *bla*_IMP-45_ were obtained from the 11 ST508 outbreak strains and HS17-127, HS18-41 from Hospital HS as well as KM18-18 and RJ19-28 from another two hospitals. All the transconjugants contained the same *guaA* allele with the recipient *P. aeruginosa* PAO1^Rif^. Transconjugants displayed similar antimicrobial susceptibility profiles with their donors except that 3 transconjugants (TcHS15-158, TcHS15-172 and TcHS15-209) were susceptible to quinolones (Supplementary Table S4). However, transfer of *bla*_IMP-45_ to *E. coli* failed.

### General features of bla_IMP-45_-harbouring plasmids

**Figure 2.**
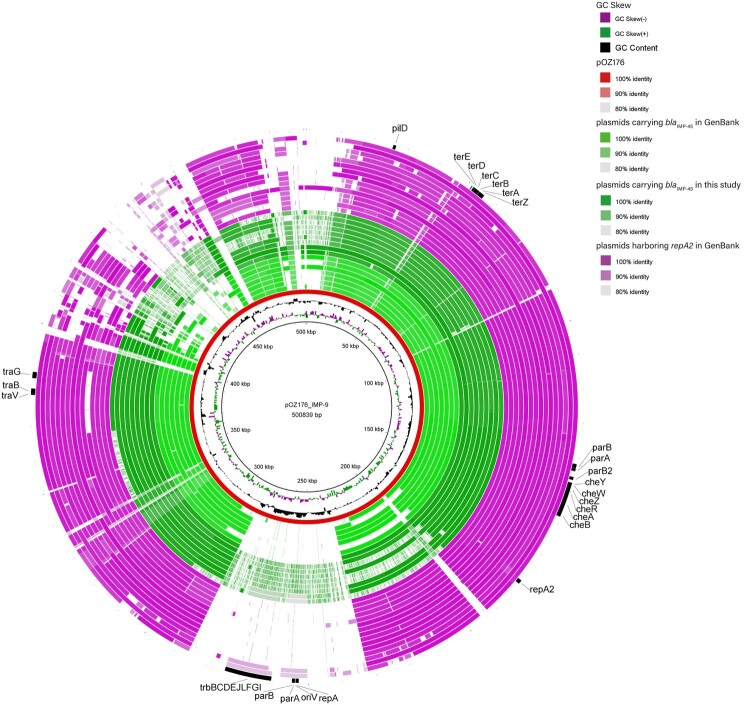
Genome comparison of plasmids containing a *repA2* gene with IncP-2 plasmid pOZ176. This map was constructed with BRIG. The various colour levels indicate a BLAST result with sequence identity ranging from 80% to 100%. The inner red circle represents the pOZ176 reference sequence. The light green, dark green and purple circles (from innermost to outermost) indicate genome sequence of 7 *bla*_IMP-45_-carrying megaplasmids in GenBank (p727-IMP, pA681-IMP, pBM413, pBM908, pPAG5, pR31014-IMP and pSY153-MDR), 9 *bla*_IMP-45_-carrying plasmids in this study (pHS17-127, pRJ19-28, pHS15-101, pHS15-106, pHS15-158, pHS15-172, pKM18-18, pHS18-41 and pGZ18-2) and 16 completely sequenced megaplasmids harbouring *repA2* in GenBank (pBT2101, pPABL048, AR441_unnamed3, AR_0356_unnamed2, RW109 plasmid 1, p12939-PER, pCF39S, pNK546-KPC, AR439_unnamed2, pRBL16, p1, pBT2436, p12969-DIM, pJB37 and pTTS12), respectively Backbone genes of pOZ176 are indicated in the figure.

*P. aeruginosa* clinical isolate HS17-127 was fully sequenced resulting in one chromosome and one plasmid pHS17-127. The sequencing analysis revealed that *repA2* and *bla*_IMP-45_ genes were on the plasmid pHS17-127 (486,963 bp), which shared a highly similar backbone with IncP-2 plasmid pOZ176 (Figure 2).

Whole genome sequences of 3 transconjugants and 5 clinical isolates in this study (Supplementary Table S5) were compared to the fully sequenced plasmid pHS17-127 and the 7 completely sequenced *bla*_IMP-45_-harbouring plasmids in GenBank (up to April, 2020). The 7 plasmids, varying in size from 374 kb to nearly 514 kb, were isolated in China and from *P. aeruginosa* except for 1 from *P. putida* [6] (Table 2). Comparative genome analysis revealed that these 16 *bla*_IMP-45_-carrying plasmids (Table 2) shared an overall similar backbone, including genes essential for replication (*repA2*, 1188 bp), partition (*par*) and conjugal transfer (*tra*) (Figure 2). Moreover, they contained an operon *terABCDEZ* conferring tellurite resistance, which is a uniform property of IncP-2 plasmids. Virulence factors, such as pilus biogenesis gene *pilD* and chemotaxis gene cluster, *cheABRWXZ*, were also identified in the backbones.

**Table 2.** Characteristics of 16 *bla*_IMP-45_-carrying plasmids (9 in this study and 7 in GenBank).

Plasmid	Host strain	ST	Location	Resistance gene*^c^*	Size (bp)^d^	Resource	Accession number ^e^
pHS15-101	*P. aeruginosa* HS15-101	ST508	Shanghai, China	*bla*_IMP-45_, *bla*_OXA-1_, *armA*, *qnrVC1*	ND	This study	N
pHS15-106	*P. aeruginosa* HS15-106	ST3014	Shanghai, China	*bla*_IMP-45_, *bla*_OXA-1_, *armA*, *qnrVC1*	ND	This study	N
pHS15-158	*P. aeruginosa* HS15-158	ST508	Shanghai, China	*bla*_IMP-45_, *bla*_OXA-1_, *armA*	ND	This study	N
pHS15-172	*P. aeruginosa* HS15-172	ST508	Shanghai, China	*bla*_IMP-45_, *bla*_OXA-1_, *armA*	ND	This study	N
pHS17-127	*P. aeruginosa* HS17-127	ST369	Shanghai, China	*bla*_IMP-45_, *bla*_OXA-1_, *bla*_PER-1_, *bla*_AFM-1_, *armA*, *qnrVC6*	486,963	This study	CP061377
pHS18-41	*P. aeruginosa* HS18-41	ST357	Shanghai, China	*bla*_IMP-45_, *bla*_OXA-1_, *armA*, *qnrVC1*	ND	This study	N
pGZ18-2	*P. aeruginosa* GZ18-2	ST1420	Guangzhou, China	*bla*_IMP-45_, *bla*_OXA-1_, *armA*, *qnrVC1*	ND	This study	N
pKM18-18	*P. aeruginosa* KM18-18	ST274	Kunming, China	*bla*_IMP-45_, *bla*_OXA-1_, *armA*, *qnrVC1*	ND	This study	N
pRJ19-28	*P. aeruginosa* RJ19-28	ST708	Shanghai, China	*bla*_IMP-45_, *bla*_OXA-1_, *bla*_AFM-1_, *qnrVC6*	ND	This study	N
pR31014-IMP	*P. aeruginosa*	unknown	China	*bla*_IMP-45_, *bla*_OXA-1_, *armA*, *qnrVC1*	374,000	GenBank	MF344571
p727-IMP	*P. aeruginosa*	unknown	China	*bla*_IMP-45_, *bla*_OXA-1_, *armA*, *qnrVC1*	430,173	GenBank	MF344568
pSY153-MDR	*P. putida* SY153	unknown	Hainan, China	*bla*_IMP-45_, *bla*_OXA-1_, *armA*, *qnrVC1*	468,170	GenBank	KY883660
pA681-IMP	*P. aeruginosa*	ST274	China	*bla*_IMP-45_, *bla*_OXA-1_, *armA*, *qnrVC6*	397,519	GenBank	MF344570
pBM908	*P. aeruginosa* PA298	ST277	China	*bla*_IMP-45_, *bla*_OXA-1_	395,774	GenBank	CP040126
pPAG5	*P. aeruginosa* PAG5	unknown	China	*bla*_IMP-45_, *bla*_OXA-1_, *armA*, *qnrVC1*	513,322	GenBank	CP045003
pBM413	*P. aeruginosa* PA121617	ST389	Guangzhou, China	*bla*_IMP-45_, *bla*_OXA-1_, *armA*, *qnrVC6*	423,017	GenBank	CP016215
^a^	*P. aeruginosa* M140A	ST308	Beijing, China	*bla*_IMP-45_, *bla*_OXA-1_	NA	GenBank	KJ510410
^b^	*P. aeruginosa* 14.1819	ST235	France	*bla*_IMP-45_, *bla*_OXA-1_, *armA*, *qnrVC1*	NA	GenBank	KU984333

^a^ *bla*_IMP-45_ was located on the chromosome.

^b^ Detailed information of plasmid carrying *bla*_IMP-45_ in *P. aeruginosa* 14.1819 was not available.

^c^ Resistance genes referring to β-lactamase genes, *armA* and *qnrVC* here.

^d^ N: Putative plasmids in this study without complete sequence. NA: Not available.

**^e^**N: Plasmids in this study without complete sequence.

### An IncP-2 plasmid sublineage associated with dissemination of bla_IMP-45_

In order to explore the mechanism underlying dissemination of *bla*_IMP-45_, 16 *bla*_IMP-45_-carrying plasmids were compared with IncP-2 *bla*_IMP-9_-harbouring plasmid pOZ176. Comparative analysis revealed that they shared similar plasmid backbones (Figure 2). However, the IncP-2 *repA*-*oriV*-*parAB* region of pOZ176 was absent in all *bla*_IMP-45_-harbouring plasmids except that pHS18-41 and pGZ18-2 contained a region with only about 84% identity. On the contrary, a second replication/partitioning system *repA2/parAB*-*parB2* was shared by both *bla*_IMP-9_- and *bla*_IMP-45_-harbouring plasmids with an identity of >98%.

**Figure 3.**
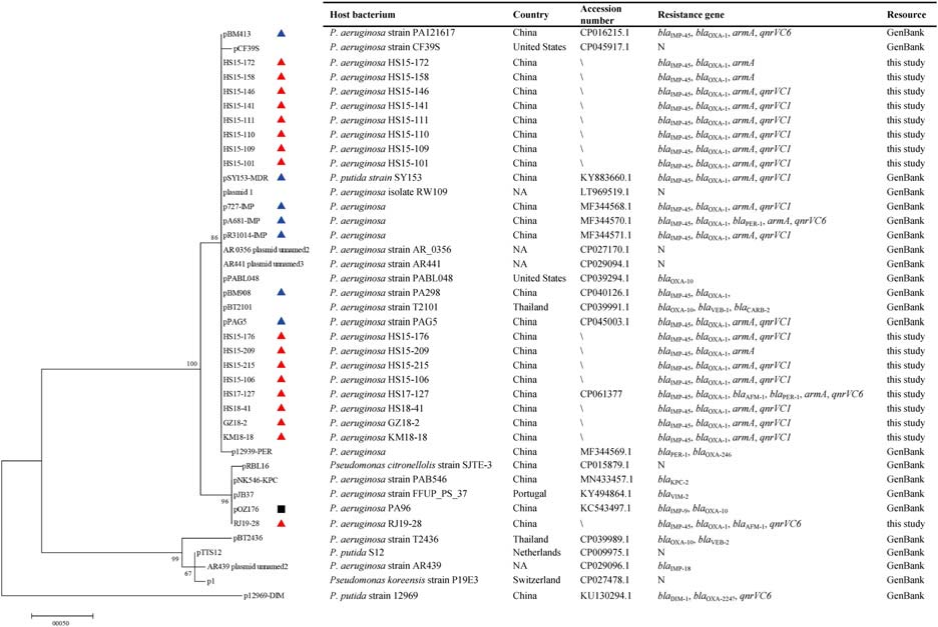
A maximum likelihood phylogenetic tree of 40 *repA2* genes and features of their hosts. Red solid triangle, blue solid triangle and black solid square indicate *bla*_IMP-45_-carrying plasmids in this study, 7 *bla*_IMP-45_-carrying megaplasmids in GenBank and IncP-2 plasmid pOZ176, respectively. Resistance genes refer to β-lactamase genes, *armA* and *qnrVC* here. “N” denotes that the plasmid harbours no β-lactamase genes, *armA* or *qnrVC*.

When BLASTN with the *repA2* gene of pOZ176 was performed for its homologs (100% query coverage), a total of 23 fully sequenced megaplasmids were identified in NCBI database, including pOZ176 and the above 7 *bla*_IMP-45_-harbouring plasmids (Figure 3). They shared a similar backbone with pOZ176 as well as the *bla*_IMP-45_-carrying plasmids in this study, even though these megaplasmids were absent of a large fragment containing *repA*-*oriV*-*parAB* of pOZ176 (Figure 2). Additionally, *repA2* genes were confirmed by PCR and sequencing in the remaining 8 transconjugants of outbreak strains without WGS data in this study (Supplementary Table S2). Phylogenetic analysis of the 40 *repA2* genes from 17 *bla*_IMP-45_-carrying CRPA in this study and 23 megaplasmids in GenBank revealed 4 distinct subgroups (Figure 3). All the *bla*_IMP-45_-carrying plasmids with the exception of RJ19-28 were in a sublineage separate from the one containing *bla*_IMP-9_-harbouring pOZ176. IncP-2 plasmids and other plasmid lineages, such as IncP-7 and IncP-9, were phylogenetically analysed on the basis of replication genes, revealing that IncP-2 plasmid lineage was clearly seperated from other plasmid lineages (Figure S2).

### Coexistence of bla_IMP-45_, arma and qnrVC1 or qnrVC6

Apart from 1 plasmid carrying only *bla*_IMP-45_ and 1 with both *bla*_IMP-45_ and *qnrVC6*, 14 out of 16 plasmids co-carried *bla*_IMP-45_ and *armA*, including 9 together with *qnrVC1* and 3 with *qnrVC6* (Table 2)*.* The genetic structures harbouring these resistant determinants were confirmed by WGS analysis or PCR and sequencing (Supplementary Table S2). As in the previously reported IMP-45 producers [5,8], *bla*_IMP-45_ was located in the variable region of In786, adjacent to a Tn*1548*-derivative containing *armA* with or without *qnrVC6* (Figure S1). qnrVC1 was in class 1 integron In1237 that coexisted with the Tn*1548*-derivative on the megaplasmids. However, In1237 was not transferred to the recipient strain together with *bla*_IMP-45_-harbouring plasmids from HS15-158, HS15-172 and HS15-209.

## Discussion

In this study, 17 IMP-45-producers were discovered and belonged to 7 dissimilar STs. All these STs were different from previously reported IMP-45-producers, such as ST308, ST235 and ST389 [5,7,8], demonstrating the diverse population structure of IMP-45-producing *P. aeruginosa*.

Outbreaks of MBL-producing *P. aeruginosa* have been reported in hospitals worldwide [23–25]. Recently, an outbreak of IMP-19-producing ST235 and IMP-29-producing ST111 of clinical *P. aeruginosa* was reported in France [26]. Here we report an outbreak caused by IMP-45-producing ST508 CRPA isolates in Hospital HS from January to September, 2015. Since around September, 2015, strengthened infection control measures were implemented in the ICU, including improved hospital-wide sanitation, hand and environmental hygiene, contact precautions, changing disinfection to sterilization for reusable ventilator accessories (exhalation valve and respiratory humidifier) and using disposable ventilator circuits instead of recycled ones. The outbreak clone subsequently disappeared and distinct clonal complex lineages carrying *bla*_IMP-45_ emerged sporadically in 2017 and 2018, suggesting a shift of the *bla*_IMP-45_-carrying CRPA clones during the survey period.

Although at least 14 incompatible groups (IncP-1∼IncP-14) of plasmids have been identified in *Pseudomonas* species, there is no well-established scheme for the plasmid typing of this species as of *Enterobacteriaceae* [27,28]. Similar to IncP-2 plasmids reported previously [9,10], the *bla*_IMP-45_-carrying plasmids are tellurite resistant, conjugative, and transfer between *P. aeruginosa* and *P. putida* but not to *E. coli*. Plasmids usually undergo continuous rearrangement and mutations, sometimes occurring in regions for plasmid typing [29], resulting in novel untypeable plasmids or new plasmid lineages evolving from currently well-studied plasmid types. The incompatibility group of *bla*_IMP-45_-carrying plasmids has not been fully clarified since they lack the region containing IncP-2 *repA*-*oriV*-*parAB* [9]. A previous study reported that IncP-2 *repA*-*oriV*-*parAB*, an auxiliary replicon, was located in an integrative and conjugative element Tn*6398a* [30]. Moreover, homologs of IncP-2 *repA* gene were seldom present in *Pseudomonas spp.* but more frequently found in putative genomic islands on the chromosome of a variety of other species, such as *Azotobacter*, *Burkholderia*, *Stenotrophomonas* and *Xanthomonas* [9]. In contrast, IncP-2 *repA2* gene and its close relatives (>96%) have been exclusively discovered on plasmids from *Pseudomonas spp.*, indicating that the *repA2* gene is probably the actual one encoding the IncP-2 replication initiator protein and contributing to the narrow-host-range of IncP-2 plasmids for *Pseudomonas spp*.

Based on the phylogenetic analysis of *repA2* and the comparative genome analysis in this study, all the plasmids involved are closely related genetically, belonging to the same plasmid family as IncP-2 pOZ176. The phylogenetic tree of *repA2* grouped almost all *bla*_IMP-45_-carrying plasmids into a different subgroup from the one containing pOZ176.

In summary, clonal diversity was observed in the 17 IMP-45-producing CRPA isolates identified in this study except for outbreak clone ST508 from Hospital HS. All the *bla*_IMP-45_-carrying plasmids were related to IncP-2 plasmid pOZ176, and contributing to the dissemination of *bla*_IMP-45_. Moreover, this IncP-2 plasmid sublineage has undergone multiple evolutionary events, recruiting *bla*_IMP-45_, *armA* and *qnrVC1/qnrVC6*, thus acting as a vehicle for the dissemination of carbapenem, aminoglycoside and quinolone resistance among *Pseudomonas spp.*, with consequent compromise of therapeutic options.

## Supplementary Material

Supplemental Material

Supplemental Material
