# Supplementary figures and images for: An IncP-2 plasmid sublineage associated with dissemination of blaIMP-45 among carbapenem-resistant Pseudomonas aeruginosa
Source: Emerg Microbes Infect. 2021 Mar 13;10(1):442–9. doi: 10.1080/22221751.2021.1894903 (PMC7971254; doi:10.1080/22221751.2021.1894903)

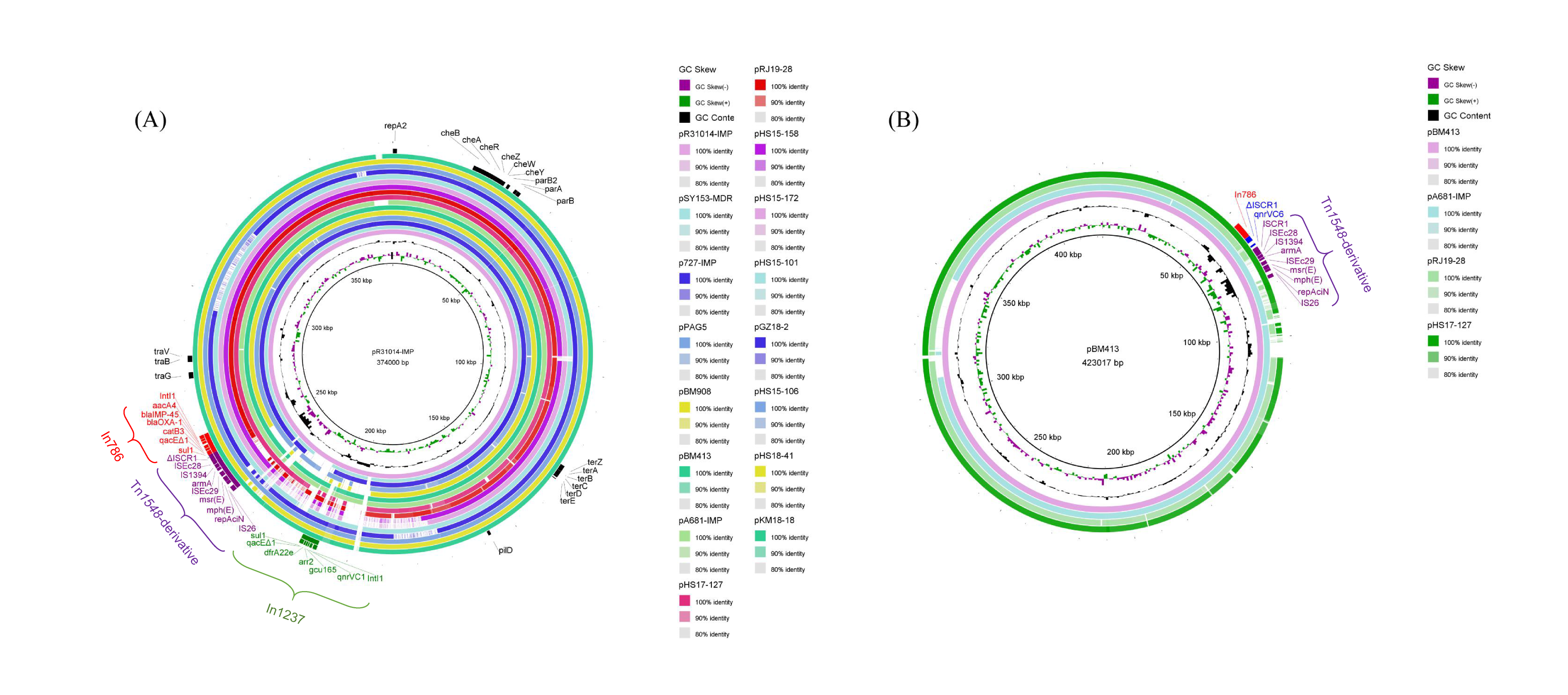

Supplement: Supplemental Material [file TEMI_A_1894903_SM4103.tif]

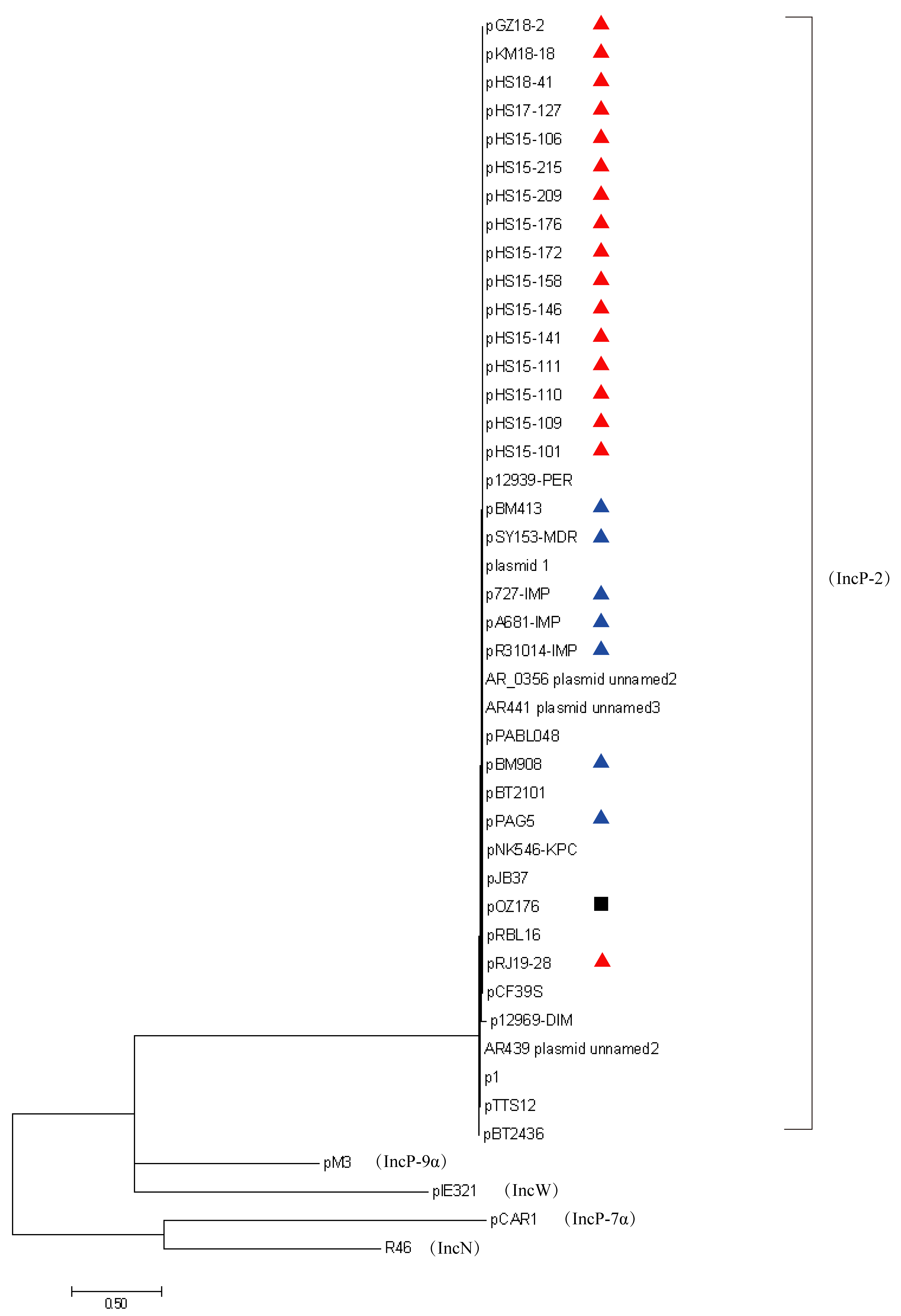

Supplement: Supplemental Material [file TEMI_A_1894903_SM0063.tif]
